# Supplementary material for: A malectin‐like receptor kinase regulates cell death and pattern‐triggered immunity in soybean
Source: EMBO Rep. 2020 Sep 14;21(11):e50442. doi: 10.15252/embr.202050442 (PMC7645207; doi:10.15252/embr.202050442)
Supplement: Supplementary file 1 — Appendix [file EMBR-21-e50442-s001.pdf]

Appendix

Appendix Table S1. Chi-square test for segregation ration of normal and mutant plants in the F2 generation (*Gmlmm1-1* × Hedou 12)…………………………………..…. (Page 1)

Appendix Table S2. INDEL molecular markers for fine-mapping……………..…(Page 1)

Appendix Table S3. Thirteen genes in the mapping region………………………(Page 2)

Appendix Table S4. Homology analysis of malectin-like RK family members on Chromosome 13 in soybean……………………………………………………….(Page 3)

Appendix Table S5. Comparison of plant height, main stem node number and other yield traits……………………………………………………………………………..(Page 3)

Appendix Table S6. Chi-square test for segregation ration of F2 generation (*Gmlmm1-1*×*Gmlmm1-2*)……………………………………………………………..(Page 3)

Appendix Table S7. Primers used in this study…………………………………..(Page 4)

Appendix Table S1. Chi-square test for segregation ration of normal and mutant plants in the F2 generation (*Gmlmm1-1* × Hedou 12)

Cross	Generation	No. of plants/lines						
		Total	Wild type	Mutant type	Expected ratio	x^2	*p*	*df*
Gmlmm1-1× Hedou 12	F2	464	372	92	3:1	3.28	0.07021	1

Appendix Table S2. INDEL molecular markers for fine-mapping

Primers	Chromosome	Physical position	Sequence 5'→3'
MOL3644&MOL3645	Gm13	9591880	TTAGCCCTGTTTACCTCAATCC
			GACCCTTCCTTTGACTGTTGC
MOL3646&MOL3647	Gm13	10007800	ACGGAAGAAAGATTGTTCATGG
			CGATGTAGGGGCAAAATGG
MOL3650&MOL3651	Gm13	13891579	AGGCTCACCTCCGCAGATAG
			CGGCACAGACTGAGCAATAGAG
MOL3652&MOL3653	Gm13	14046684	AGAAAGCCTATGTCGGCAAGA
			TCAAAGGGTGGTCCTTCACA
MOL3654&MOL3655	Gm13	15477038	CATCCACCATGAAAATTAACCC
			CAATCATCAAAATCCCATCCCT
MOL3678&MOL3679	Gm13	14537849	CCAAGGGACTGTGAGGTGAAC
			GGATGGACAACTCCAAGAAGAA
MOL36807MOL3681	Gm13	14653018	ATGCACTCGTTTGCACAATTC
			GCTTCTGCATTGACATGACGC
MOL3682&MOL3683	Gm13	14778920	TGCGAGATCCAATGTGGTTAC
			TGTAGGTTGCGTCTCGTTTGT
MOL3684&MOL3685	Gm13	15061138	TACATGGAGCGTGCTTATCAAA
			TGTTGACCAATATGCGTGTCTT
MOL3686&MOL3687	Gm13	15508918	GGGAGATTTCACATCAAGTTCG
			TGAGGATGGGAAACCAGTCAGT
MOL3688&MOL3689	Gm13	16048790	TCCCTGTTTGGCTTCAGTCTAC
			CGGAGCTGTTGCACCTTTGT
MOL3692&MOL3693	Gm13	16787574	TGGTCGCATGACATCATAACAA
			CATTGGCTGGAATGAGAAGC
MOL3754&MOL3755	Gm13	15077108	CAAGAAGCACCCCACCCA
			CGATGAAGACGGTGACAGTGAG
MOL3756&MOL3757	Gm13	15131757	GTCGTGGTTCGCAAGAAAGC
			TGGCCTCGTTGAGCAAATC
MOL3758&MOL3759	Gm13	15231494	CCAAAAGGAGCAGAAAGTCAAG
			TTGAGTGAAGCCAAGGAGGAG
MOL3760&MOL3761	Gm13	15233406	CTCAATCTCCCTTACCCCTTTT

			GTGGGAACTCGTTCTGTATCGT
MOL3770&MOL3771	Gm13	15133035	AAGGAGCGGTATGAGGGAGC
			CAATGCCACTGCACAAGAAAC
MOL3772&MOL3773	Gm13	15158889	TTCTAAAACCCTCCCACCCC
			CAGTTGGGTGTATTTGAAGGCT
MOL3774&MOL3775	Gm13	15160087	GCCTGATAGATGCGACAAAGTT
			GACCTTAAATGCACGTCGTTG
MOL3776&MOL3777	Gm13	15221786	TGGATTGCTTAGTTCCAAGACG
			CCAAATCGTTGCCAATAAGG
MOL3778&MOL3779	Gm13	15188188	GCCACTGCTTTTGAGTGGATG
			AATGCCAAAGGTGAATCGTG
MOL3780&MOL3781	Gm13	15208153	TTTTCCCTTAAAAGGTAAATCC
			GGTTCAATAGTCAAGCTCGTTG

Appendix Table S3. Thirteen genes in the mapping region

**Genes**	**Protein family**
Glyma.13G053500	No annotation
Glyma.13G053600	Malectin/receptor-like protein kinase family protein
Glyma.13G053700	Malectin/receptor-like protein kinase family protein
Glyma.13G053800	Malectin/receptor-like protein kinase family protein
Glyma.13G053900	Protein kinase superfamily protein
Glyma.13G054000	HXXXD-type acyl-transferase family protein
Glyma.13G054100	GAST1 protein homolog 1
Glyma.13G054200	Malectin/receptor-like protein kinase family protein
Glyma.13G054300	Malectin/receptor-like protein kinase family protein
Glyma.13G054400	Malectin/receptor-like protein kinase family protein
Glyma.13G054500	ARM repeat superfamily protein
Glyma.13G054600	Pectin lyase-like superfamily protein
Glyma.13G054700	No annotation

Appendix Table S4. Homology analysis of malectin-like RK family members on Chromosome 13 in soybean

Members of MRLK Family on Chromosome 13 in Soybean			
**Gene ID**	**Protein Length (aa)**	**Identities**	**E-value**
Glyma.13G054400 (GmLMM1)	896	-	-
Glyma.13G053600	894	98.55%	0
Glyma.13G053800	702	73.80%	0
Glyma.13G054300	844	80.53%	0
Glyma.13G053700	819	79.75%	0
Glyma.13G054200	787	67.22%	0

Appendix Table S5. Comparison of plant height, main stem node number and other yield traits.

**Genotype**	**Plant height; cm**	**Main stem node number**	**Single seed pod**	**Two seed pod**	**Three seed pod**	**Grain weight per plant; g**
Williams82 n=27	120.41 ±7.16	26.85 ±1.49	27.78 ±11.01	33.56 ±15.89	28.33 ±10.94	33.11 ±12.84
Gmlmm1-2 n=32	86.06± 7.32***	23.91± 2.51***	59.00± 21.62***	52.75± 19.06***	14.66± 7.42***	32.96± 11.90

Adult plants grown in field were used for measurements. Values given are means ± SD (***, $P < 0.001$, Student's *t*-test at).

Appendix Table S6. Chi-square test for segregation ration of F2 generation (*Gmlmm1-1×Gmlmm1-2*)

**Cross**	**Generation**	**No. of plants/lines**							
		**Total**	***Gmlmm1-1* gene type**	***Gmlmm1-1× Gmlmm1-2* gene type**	***Gmlmm1-2* gene type**	**Expected ratio**	x^2	***p***	***df***
Gmlmm1-1× Gmlmm1-2	F2	33	11	16	6	1:2:1	0.75581	0.6853	2

Appendix Table S7. Primers used in this study

Primers	sequence
GmLMM1-Nluc-F	acgggggacgagctcggtaccATGAGGCTCCTTAGCATCATCAC
GmLMM1-Nluc-R	aacatcgtatgggtagtcgacACGTGGCTTTGGATCCACAA
GmLMM1-CCluc-F	acgggggacgagctcggtaccATGAGGCTCCTTAGCATCATCAC
GmLMM1-CCluc-R	cgcgtacgagatctggtcgacACGTGGCTTTGGATCCACAA
Glyma.05G198700Nluc-F	acgggggacgagctcggtaccATGGGAGGCACTTCTGCAGA
Glyma.05G198700Nluc-R	aacatcgtatgggtagtcgacGAAATTTTCTTTATGGAAGTCGTACTTG
Glyma.17G226700Nluc-F	acgggggacgagctcggtaccATGCTGTCTTTTGTAACAGAAAATATGG
Glyma.17G226700Nluc-R	aacatcgtatgggtagtcgacTAACAAGCCAGCTTCCAAGAGAT
Glyma.08G083300Nluc-F	acgggggacgagctcggtaccATGTTGTCCCTAAAGATTAGTTTGACTAT
Glyma.08G083300Nluc-R	aacatcgtatgggtagtcgacACAAGACAGTGTTGTTTGAAGCTTCA
Glyma.20G008000Nluc-F	acgggggacgagctcggtaccATGGATTCTTCTGTGCCTCCTG
Glyma.20G008000Nluc-R	aacatcgtatgggtagtcgacAGGTAGTGCATCTACTACCATCCTCA
Glyma.15G051600Nluc-F	acgggggacgagctcggtaccATGGAGAGAGTGACTTCATCTTTTATGG
Glyma.15G051600Nluc-R	aacatcgtatgggtagtcgacTCTAGGACCTGATAGTTCATCTGCC
Glyma.08G180800Nluc-F	acgggggacgagctcggtaccATGGATAGAGTGACTTCGTCTTTTATG
Glyma.08G180800Nluc-R	aacatcgtatgggtagtcgacTCTAGGACCTGATAGTTCATCTGCC
Glyma.11G026400Nluc-F	acgggggacgagctcggtaccATGAGTTCCAAAGAGAGTTGCAGA
Glyma.11G026400Nluc-R	aacatcgtatgggtagtcgacGGGAGGAAAATGCTCAACATCC
Glyma.19G233900Nluc-F	acgggggacgagctcggtaccATGGAGATTCAGTTAGAGCAGCAGC
Glyma.19G233900Nluc-R	aacatcgtatgggtagtcgacAAAATTCTCTTTATGAAAATCAAACTTGG
Glyma.12G043900Nluc-F	acgggggacgagctcggtaccATGTCTGTTACGGAGCTCAAAGAGC
Glyma.12G043900Nluc-R	aacatcgtatgggtagtcgacAGTTGTCCTCCTATGCCCTCCA
NbRXEG1-Nluc-F	acgggggacgagctcggtaccATGGGCAAAAGGGAATATCCA
NbRXEG1-Nluc-R	aacatcgtatgggtagtcgacAGCCCTTAACTTTCTCTTCAGTCTTG
NbFLS2-Nluc-F	acgggggacgagctcggtaccATGTCACAGACAGTTTTATATGCATTAGC
NbFLS2-Nluc-R	aacatcgtatgggtagtcgacAATGCAGGCATACTGGCAAATC
NbBAK1-Nluc-F	acgggggacgagctcggtaccATGGATCAATGGATATTGGGGA
NbBAK1-Nluc-R	aacatcgtatgggtagtcgacTCTTGGCCCTGATAACTCATCAG
NbBAK1-FLAG-F	acgggggacgagctcggtaccATGGATCAATGGATATTGGGGA
NbBAK1-FLAG-R	atggtctttgtagtcgtcgacTCTTGGCCCTGATAACTCATCAG
NbFLS2-FLAG-F	acgggggacgagctcggtaccATGTCACAGACAGTTTTATATGCATTAGC
NbFLS2-FLAG-R	atggtctttgtagtcgtcgacAATGCAGGCATACTGGCAAATC
NbRXEG1-FLAG-F	acgggggacgagctcggtaccATGGGCAAAAGGGAATATCCA
NbRXEG1-FLAG-R	atggtctttgtagtcgtcgacAGCCCTTAACTTTCTCTTCAGTCTTG
Glyma.08G083300-FLAG-F	acgggggacgagctcggtaccATGTTGTCCCTAAAGATTAGTTTGACTAT
Glyma.08G083300-FLAG-R	atggtctttgtagtcgtcgacACAAGACAGTGTTGTTTGAAGCTTCA
Glyma.15G051600-FLAG-F	acgggggacgagctcggtaccATGGAGAGAGTGACTTCATCTTTTATGG
Glyma.15G051600-FLAG-R	atggtctttgtagtcgtcgacTCTAGGACCTGATAGTTCATCTGCC
Glyma.08G180800-FLAG-F	acgggggacgagctcggtaccATGGATAGAGTGACTTCGTCTTTTATG
Glyma.08G180800-FLAG-R	atggtctttgtagtcgtcgacTCTAGGACCTGATAGTTCATCTGCC
NbFLS2-HA-F	acgggggacgagctcggtaccATGTCACAGACAGTTTTATATGCATTAGC
NbFLS2-HA-R	aacatcgtatgggtagtcgacAATGCAGGCATACTGGCAAATC

GmLMM1-HA-F	acgggggacgagctcggtaccATGAGGCTCCTTAGCATCATCAC
GmLMM1-HA-R	aacatcgtatgggtagtcgacACGTGGCTTTGGATCCACAA
GmLMM1-GFP-F	acgggggacgagctcggtaccATGAGGCTCCTTAGCATCATCAC
GmLMM1-GFP-R	acgggggacgagctcggtaccATGAGGCTCCTTAGCATCATCAC
GmLMM1^{L407H}-GFP-F	acgggggacgagctcggtaccATGAGGCTCCTTAGCATCATCAC
GmLMM1^{L407H}-GFP-R	acgggggacgagctcggtaccATGAGGCTCCTTAGCATCATCAC
GmLMM1-FLAG-F	agaacacgggggacgagctcggtaccATGAGGCTCCTTAGCATCATCAC
GmLMM1-FLAG-R	tggtctttgtagtcgtcgacACGTGGCTTTGGATCCACAA
BIK1-HIS-F	taagaaggagatataccatggGTTCTTGCTTCAGTTCTCGAGTCA
BIK1-HIS-R	gtggtggtggtggtgctcgagCACAAGGTGCCTGCCAAAA
GmLMM1KD-HIS-F	taagaaggagatataccatggCCGCGCGTGGACTGCATT
GmLMM1KD-HIS-R	gtggtggtggtggtgctcgagAACGACGTCGTTCATCGACG
CRISPR 9 -F	ATTGCGAGGCACTGCTGCCAAAA
CRISPR 9 -R	AACTTTTGGCAGCAGTGCCTCGC
CRISPR 1 -F	ATTGATCTTTCAAGCAACACGGA
CRISPR 1 -R	AACTCCGTGTTGCTTGAAAGATC
CRISPR 2 -F	ATTGGAAACCGCAGCAGCAAAAG
CRISPR 2 -R	AACCTTTTGCTGCTGCGGTTTCC
CRISPR-R	GATGAAGTGGACGGAAGGAAGGAG
BlpR-F	ATGACAGCGACCACGCTCTTGAAG
BlpR-R	CTGCACCATCGTCAACCACTACATC
gDNA-GmLMM1-F	GAGGGAAAAGCAGGTTATTG
gDNA-GmLMM1-R	GCCAATTACGTTGCTGTTG
gDNA-Glyma.13G053600-F	TTTAAGGCAATTTAGGTGGG
gDNA-Glyma.13G053600-R	TTCTGGAACTGTTTGAGGCT
gDNA-Glyma.13G053800-F	GATTCCACCTTGATCTTCCTAA
gDNA-Glyma.13G053800-R	AAGTGTAACATGACTCCCCATA
GmELF1β-F	GTTGAAAAGCCAGGGGACA
GmELF1β-R	TCTTACCCCTTGAGCGTGG
GmPR1-F	ACCGGTAACCTAAGTGGTACAAATG
GmPR1-R	CCACATCCAAGACGCACAGAGT
GmPR2-F	GTCTCCTTCGGTGGTAGTG
GmPR2-R	ACCCTCCTCCTGCTTTCTC
PsTEF1-F	TGATCGTGCTGAACCACCC
PsTEF1-R	CGAGCGACGGTCCATCTT
AvrB-kpnI-F	CGGGGGACGAGCTCGGTACCATGGGCTGCGTCTCGTCAAA
AvrB-SalI-R	TGGTCTTTGTAGTCGTCGACTTAAAAGCAATCAGAATCTA
AvrRpt2-kpnI-F	CGGGGGACGAGCTCGGTACCATGAAAATTGCTCCAGTTGC
AvrRpt2-SalI-R	TGGTCTTTGTAGTCGTCGACTTAGCGGTAGAGCATTGCGT
