# Supplementary material for: A malectin‐like receptor kinase regulates cell death and pattern‐triggered immunity in soybean
Source: EMBO Rep. 2020 Sep 14;21(11):e50442. doi: 10.15252/embr.202050442 (PMC7645207; doi:10.15252/embr.202050442)
Supplement: Supplementary file 2 — Expanded View Figures PDF [file EMBR-21-e50442-s002.pdf]

Expanded View Figures

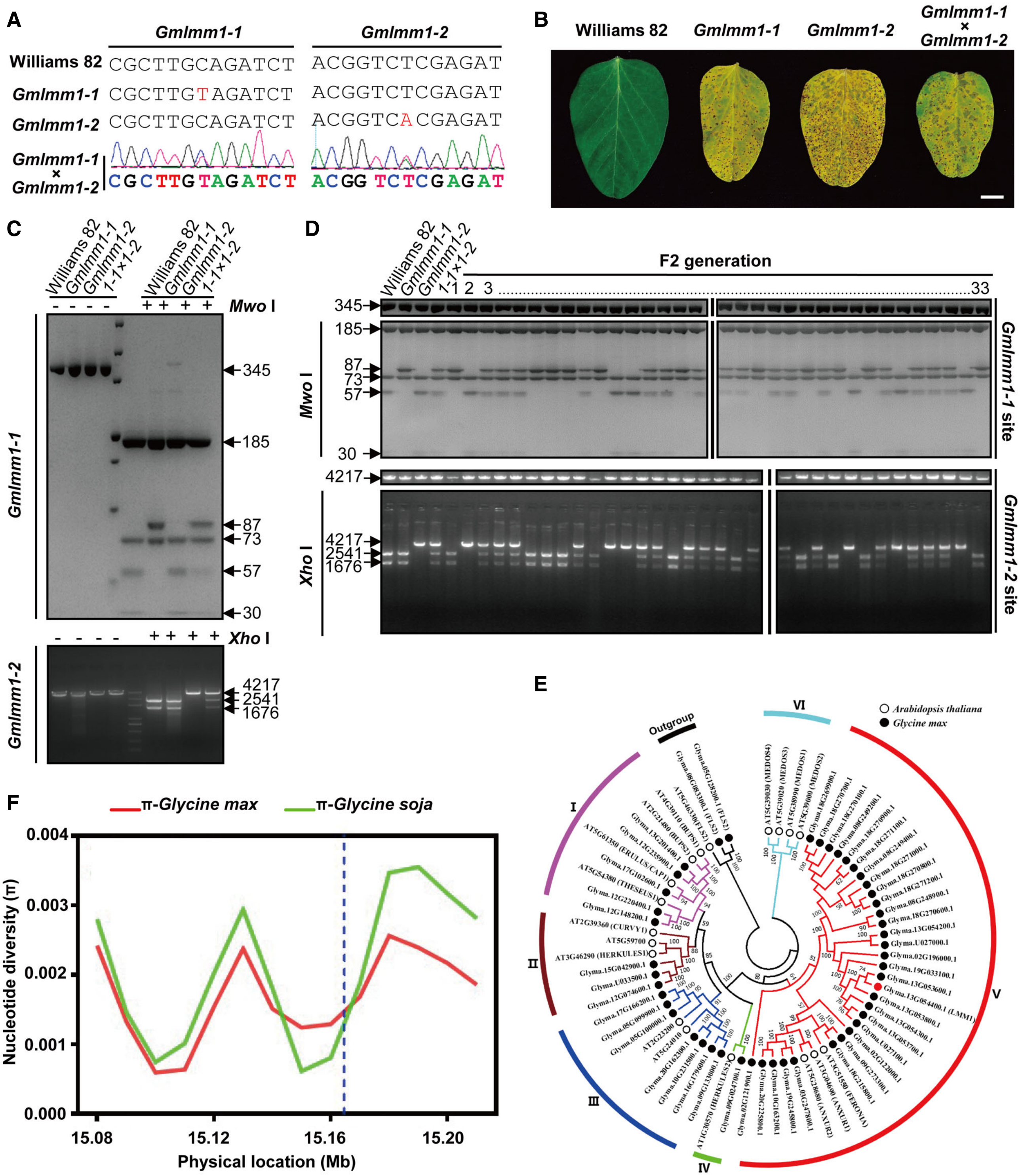

Figure EV1.

© 2020 The Authors EMBO reports e50442 | 2020 17 of 16

Figure EV1. Identification of the *Gmlmm1* mutants and bioinformatics analysis of the *GmLMM1* gene.

A Verification of the mutation site of *Gmlmm1-1* and *Gmlmm1-2* by PCR and sequencing.
B Typical phenotypes of the plants. The indicated plants were grown for 18 days in a climate chamber. Scale bar, 1 cm.
C Identification of the F1 plant (*Gmlmm1-1* × *Gmlmm1-2*) genotype by PCR and restriction enzyme digestion. Fragments covering the *Gmlmm1-1* and *Gmlmm1-2* mutation sites were amplified by PCR, digested by the indicated enzymes, and separated on agarose gel. The corresponding fragment sizes after cleavage are marked with arrows.
D Identification of the F2 (*Gmlmm1-1* × *Gmlmm1-2*) genotype. Total DNA was extracted from 33 individual F2 plants, and fragments covering the *Gmlmm1-1* and *Gmlmm1-2* mutation sites were amplified.
E Phylogenetic analysis of the malectin-like RK family in *Arabidopsis* and *Glycine max*.
F Nucleotide diversity (π) of *Glycine max* (red line) and *Glycine soja* (green line) across a ~150 kb genomic region that harbored the 13 mapped genes. The blue dotted line indicates the location of the *GmLMM1* gene. The abscissa represents the physical location (Mb) of differential nucleotides. The ordinate represents nucleotide diversity (π).

Data information: The experiments were performed three times (A, B) or two times (C, D), as biological replicates, with similar results.

Source data are available online for this figure.

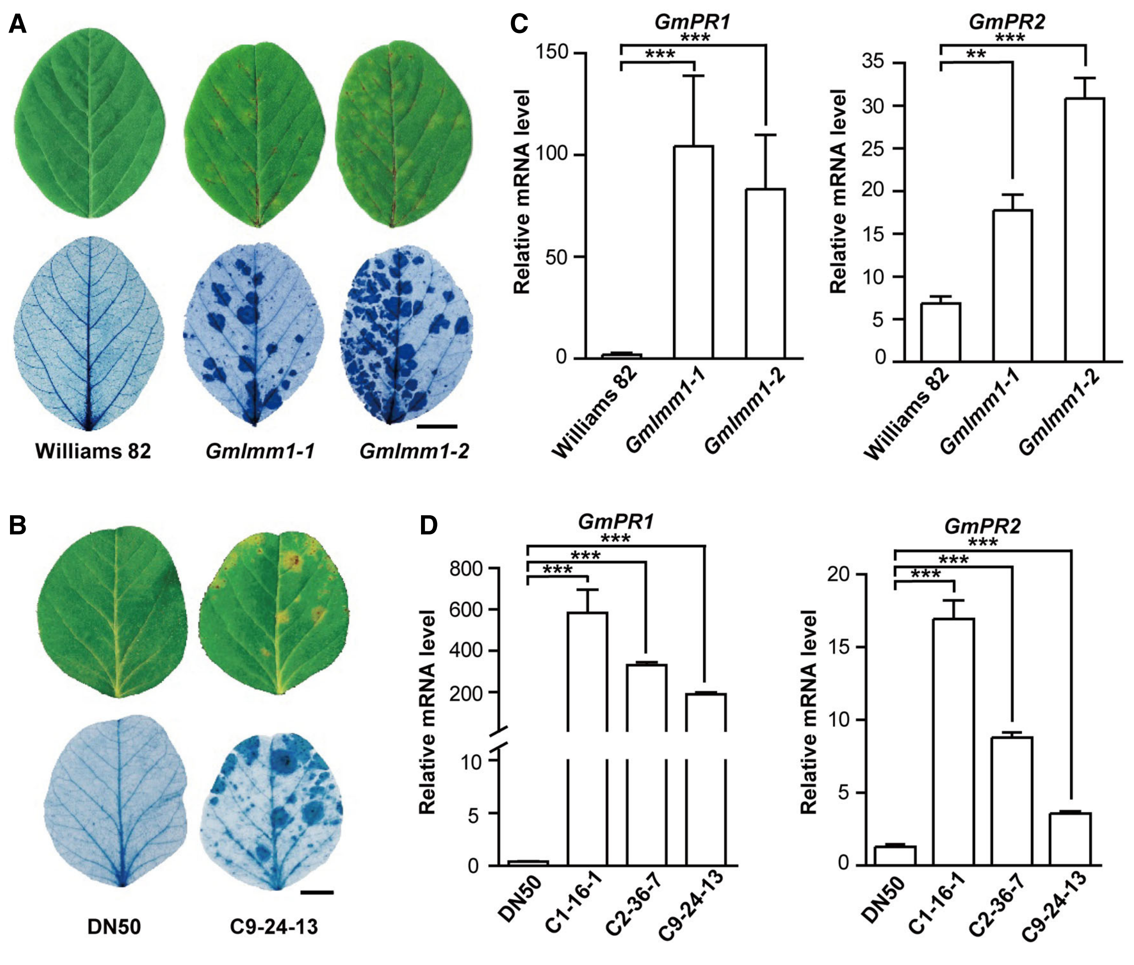

Figure EV2. *Gmlmm1* mutants are autoimmune-related lesion mimic mutants.

A Cell death examination in *Gmlmm1-1*, *Gmlmm1-2*, and Williams 82. Leaves of the indicated plants (aged 2 weeks) were subjected to trypan blue staining. Scale bar, 1 cm.
B Cell death examination in DN50 (Dongnong 50) and the CRISPR line C9-24-13. Scale bar, 1 cm.
C Expression of the defense marker genes in *Gmlmm1* mutants. *GmPR1* and *GmPR2* were examined by qPCR analysis in the indicated plants (Mean ± SD, $^{**}P < 0.01$, $^{***}P < 0.001$, Student's *t*-test).
D Expression of *GmPR1* and *GmPR2* in DN50 and the CRISPR lines. (Mean ± SD, $^{***}P < 0.001$, Student's *t*-test).

Data information: The experiments were performed three times (A, B) or two times (C, D), as biological replicates, with similar results.

 © 2020 The Authors

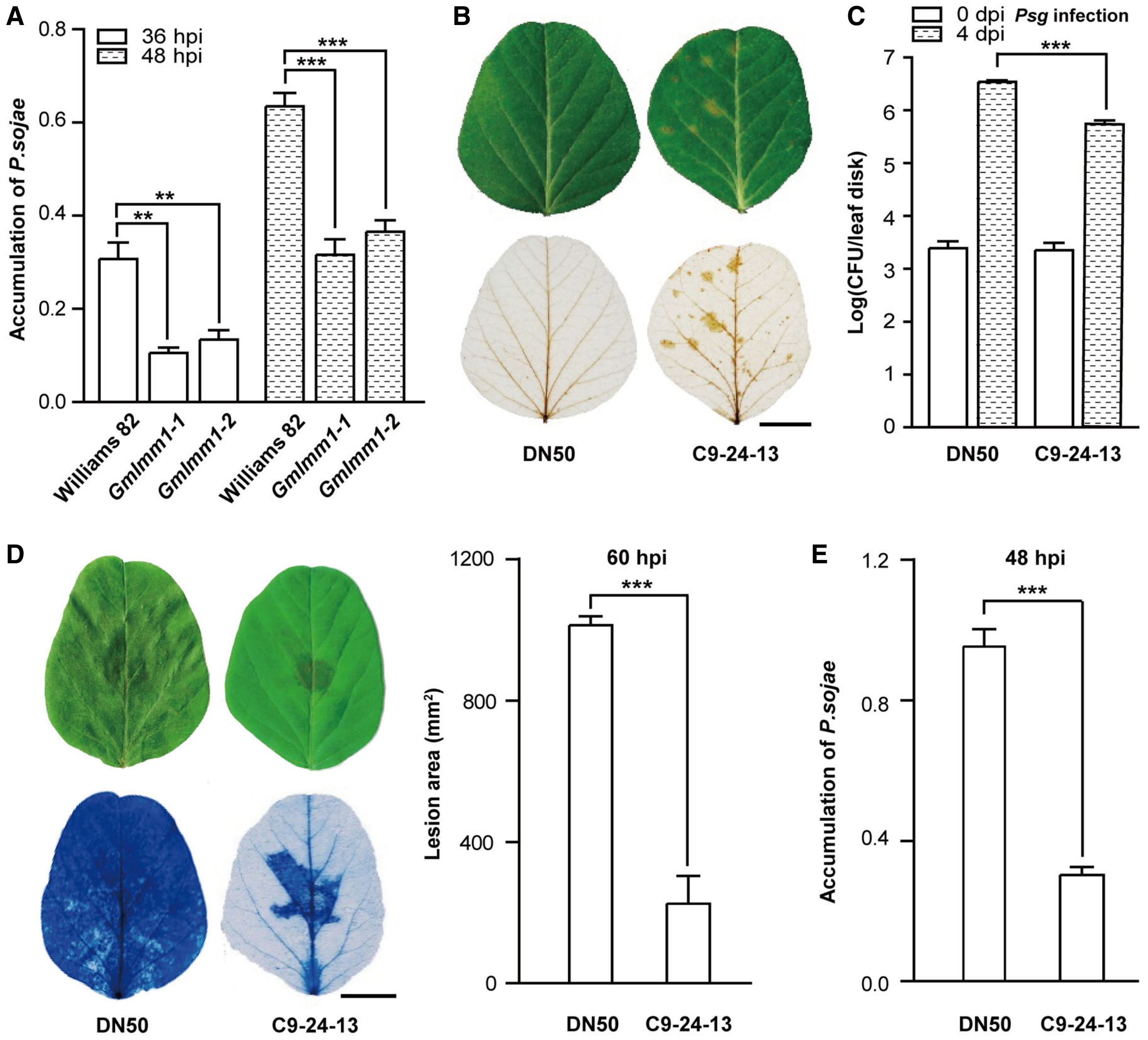

Figure EV3. The mutants are more resistant to *Psg* and *P. sojae*.

A Measurement of *P. sojae* biomass. (Mean ± SD, $^{**}P < 0.01$, $^{***}P < 0.001$, Student's *t*-test).

B ROS accumulation in DN50 and C9-24-13. Soybean leaves (aged 2 weeks) were subjected to ROS accumulation examination by DAB staining. Scale bar, 1 cm.

C The C9-24-13 line was more resistant to *Psg* infection. Scale bar, 1 cm. (Mean ± SD, $n \geq 8$, n represents sample number, $^{***}P < 0.001$, Student's *t*-test).

D The increased resistance of the C9-24-13 line to *P. sojae* infection. The indicated plants (aged 2 weeks) were infected with *P. sojae* isolate P7076, and lesions are shown by trypan blue staining at 60 hpi (left panel). Scale bar, 1 cm. The lesion areas were calculated and compared (right panel) (Mean ± SD, $n = 6$, n represents sample number, $^{***}P < 0.001$, Student's *t*-test).

E Measurement of *P. sojae* biomass (Mean ± SD, $^{***}P < 0.001$, Student's *t*-test).

Data information: All the experiments were performed three times (biological replicates) with similar results.

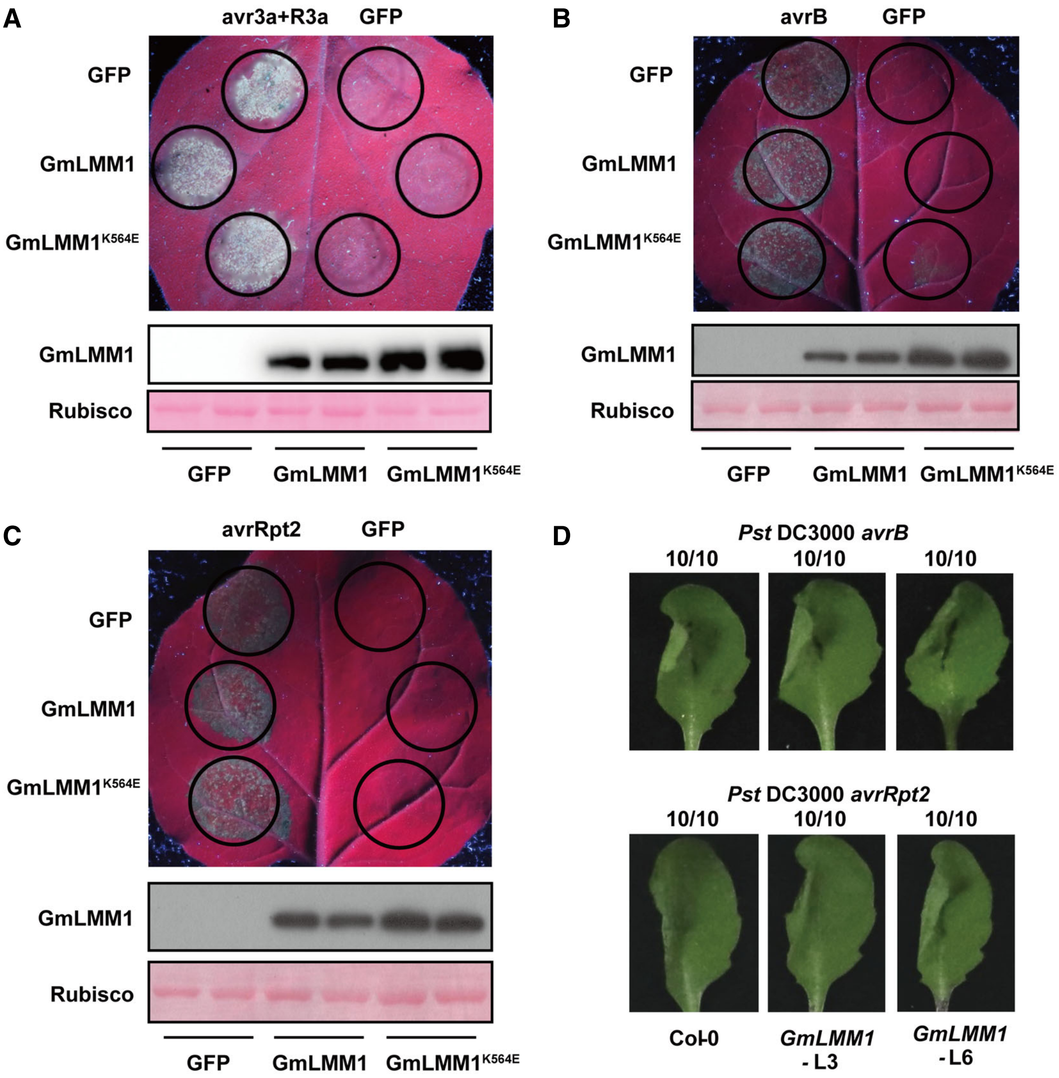

Figure EV4. Effect of GmLMM1 on effector-induced cell death.

A R3a and avr3a-induced cell death was not affected by GmLMM1. The indicated constructs were transiently expressed in *N. benthamiana* by *Agrobacterium*-mediated transient expression. The cell death phenotype was photographed under UV light. Protein expression of GmLMM1-HA and GmLMM1^{K564E}-HA is shown in the lower panel.

B, C AvrB (B) and AvrRpt2 (C)-induced cell death was not affected by GmLMM1 in *N. benthamiana*. Protein expression of GmLMM1-HA and GmLMM1^{K564E}-HA is shown in the lower panel.

D AvrB and AvrRpt2-induced cell death was not affected by GmLMM1 in Arabidopsis. *P. syringae* DC3000 carrying *avrB* or *avrRpt2* was infiltrated into the left side of the transgenic leaves expressing the *GmLMM1* gene. Cell death was scored 3 hpi (avrB) and 8 hpi (avrRpt2). Numbers indicate the ratio of leaves developing cell death and total number of inoculated leaves.

Data information: The experiments were performed three times (A) or two times (B–D), as biological replicates, with similar results.

Source data are available online for this figure.

 © 2020 The Authors

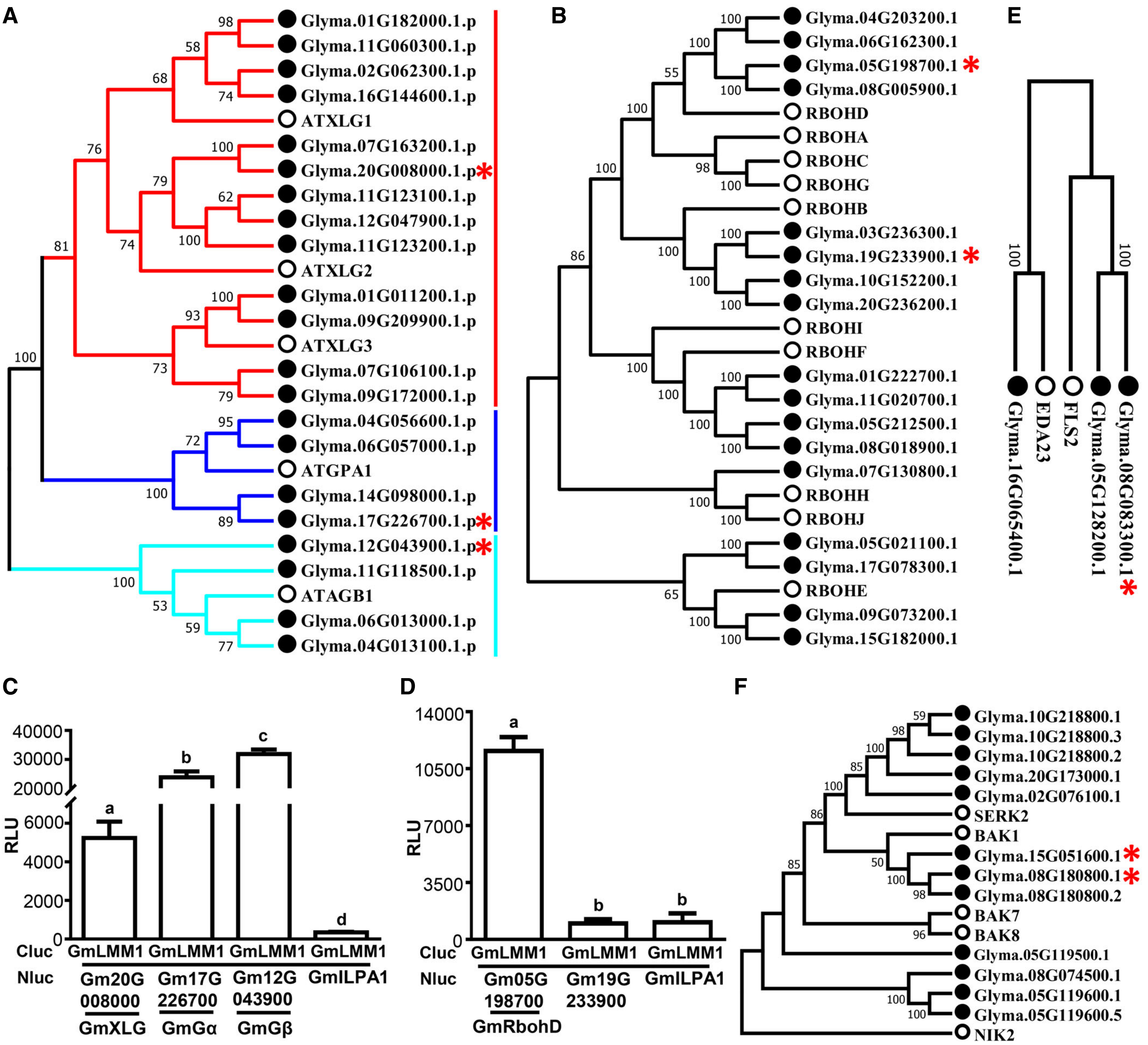

Figure EV5. GmLMM1 interacts with soybean G proteins and NADPH oxidases and phylogenetic analysis for homologs of Arabidopsis in soybean.

A Phylogenetic analysis of homologs of Arabidopsis G proteins (Gα, Gβ, and extra large G protein) in soybean. The proteins used below (C) are marked by red asterisks.

B Phylogenetic analysis of NADPH oxidases in Arabidopsis and soybean. The proteins the below (D) are marked by red asterisks.

C GmLMM1 interacts with soybean G proteins. The indicated constructs were transiently expressed in *N. benthamiana* leaves and subjected to luciferase complementation assay. Gm20G008000 (GmXLG), Gm17G226700 (GmGα), and Gm12G043900 (GmGβ) were determined (Mean ± SD, $n \geq 6$, n represents sample number, $P < 0.05$, Student's *t*-test, different letters indicate significant difference).

D GmLMM1 interacts with soybean NADPH oxidases. The indicated constructs were transiently expressed in *N. benthamiana* leaves and subjected to luciferase complementation assay. Gm05G198700 (GmRbohD) is the closest homolog of Arabidopsis RbohD. Gm19G233900 is the closest homolog of Arabidopsis RbohB (Mean ± SD, $n \geq 6$, n represents sample number, $P < 0.05$, Student's *t*-test, different letters indicate significant difference).

E Phylogenetic analysis of homologs of Arabidopsis FLS2 proteins in soybean. The proteins used in this study are marked by red asterisks.

F Phylogenetic analysis of homologs of Arabidopsis BAK1 proteins in soybean. The proteins used in this study are marked by red asterisks.

Data information: The experiments (C, D) were performed three times (biological replicates).

© 2020 The Authors
