# Supplementary figures and images for: A malectin‐like receptor kinase regulates cell death and pattern‐triggered immunity in soybean
Source: EMBO Rep. 2020 Sep 14;21(11):e50442. doi: 10.15252/embr.202050442 (PMC7645207; doi:10.15252/embr.202050442)

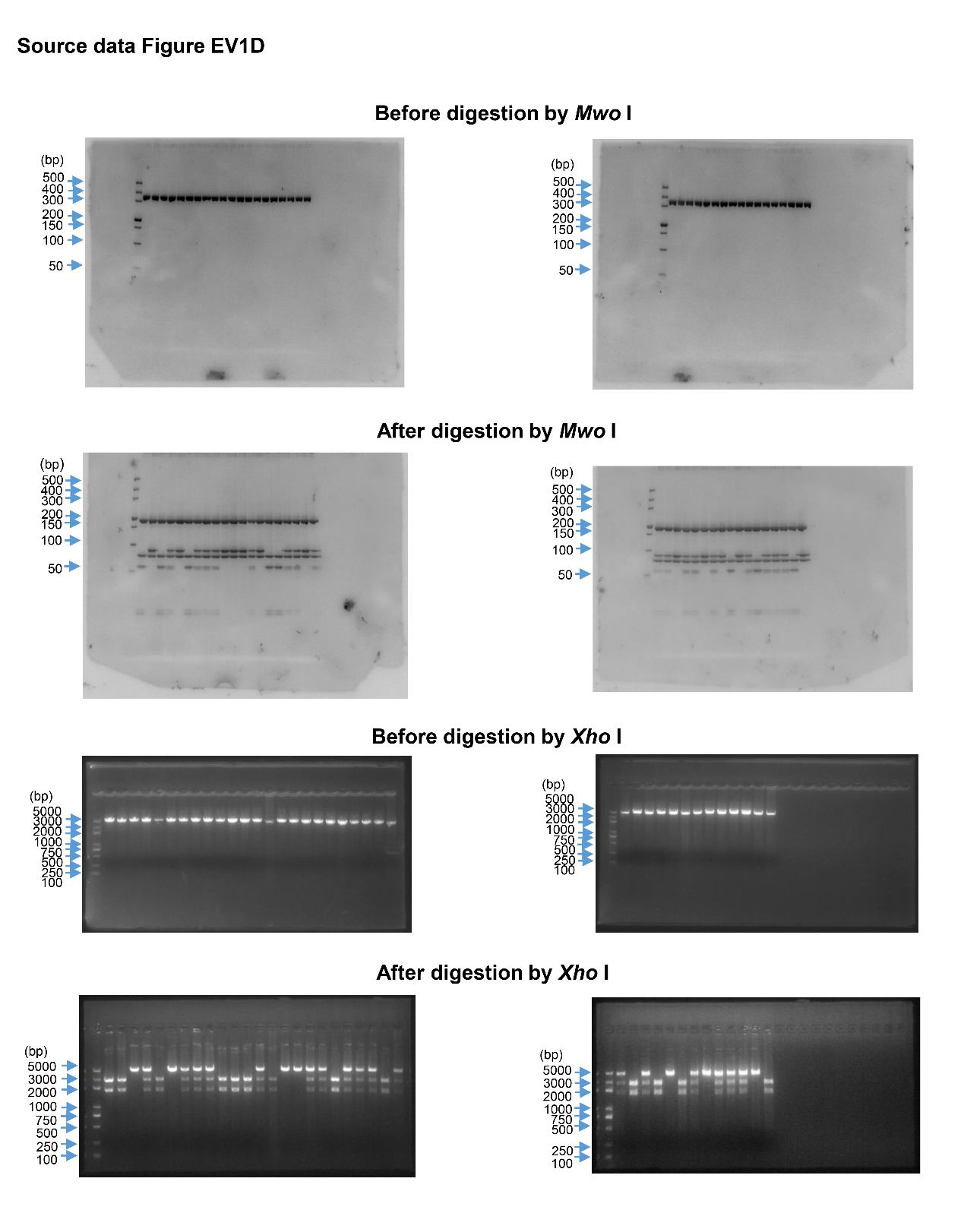

Supplement: Supplementary file 5 — Source Data for Expanded View [file EMBR-21-e50442-s010.zip › embr202050442-sup-0010-SDataFigEV/embr202050442-sup-0010-SDataFigEV1.docx]

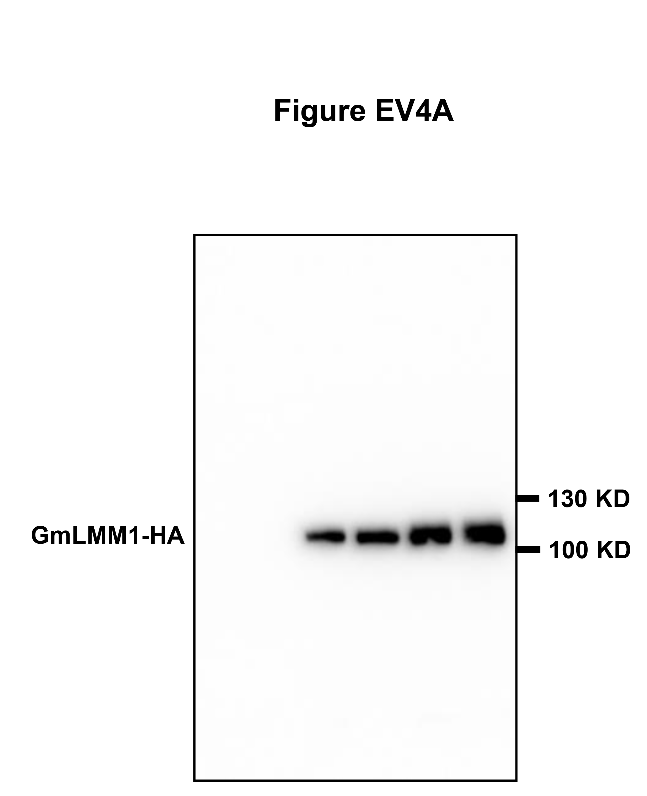


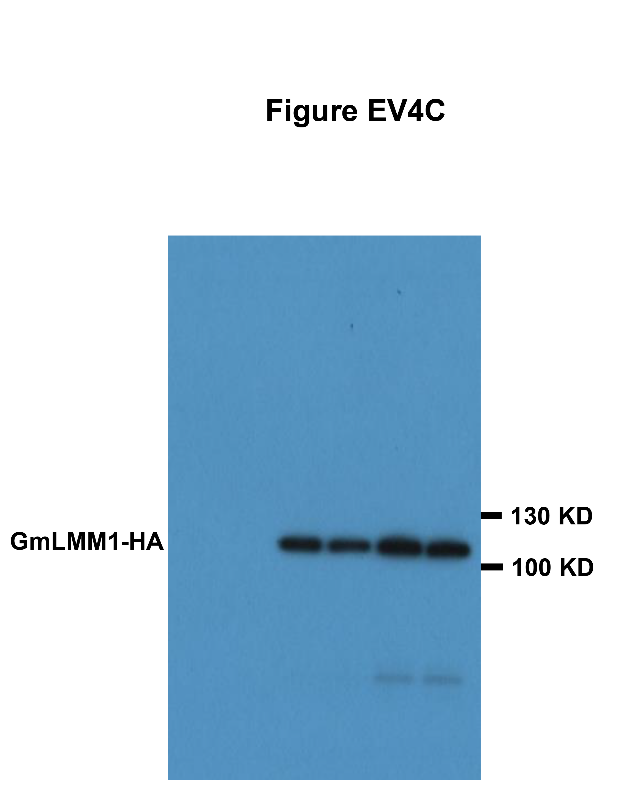

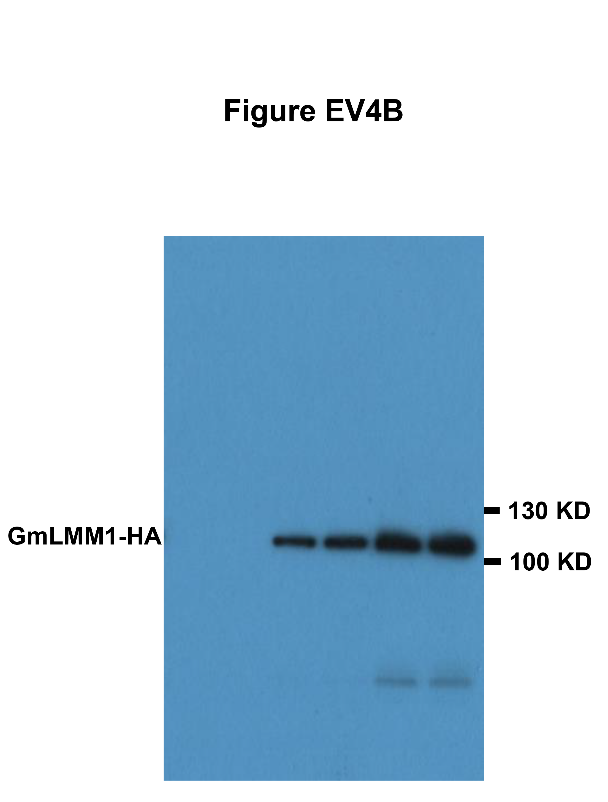

Supplement: Supplementary file 5 — Source Data for Expanded View [file EMBR-21-e50442-s010.zip › embr202050442-sup-0010-SDataFigEV/embr202050442-sup-0011-SDataFigEV4.docx]

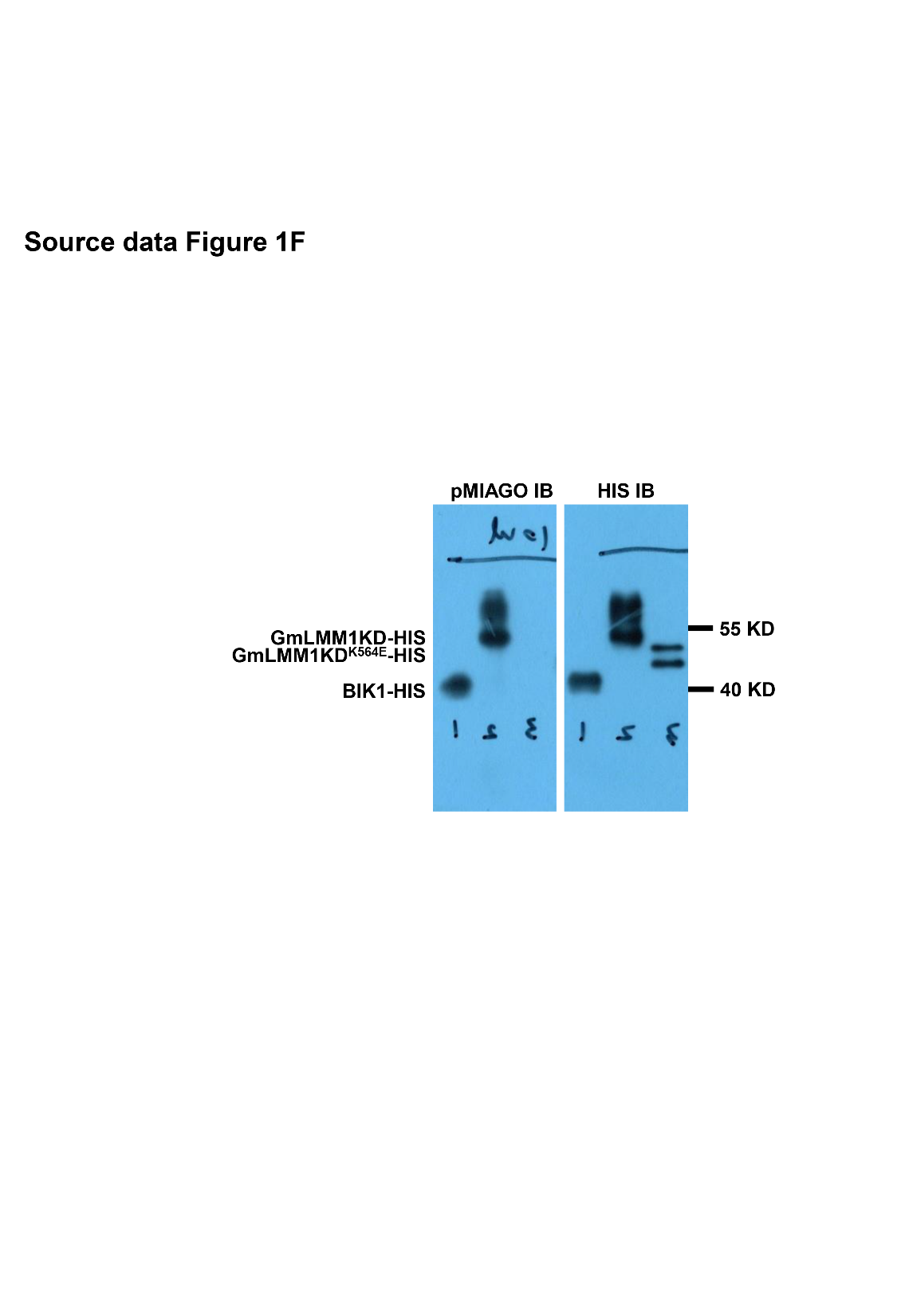

Supplement: Supplementary file 7 — Source Data for Figure 1 [file EMBR-21-e50442-s005.zip › Source Data for Figure 1/Source Data for Figure 1F.docx]

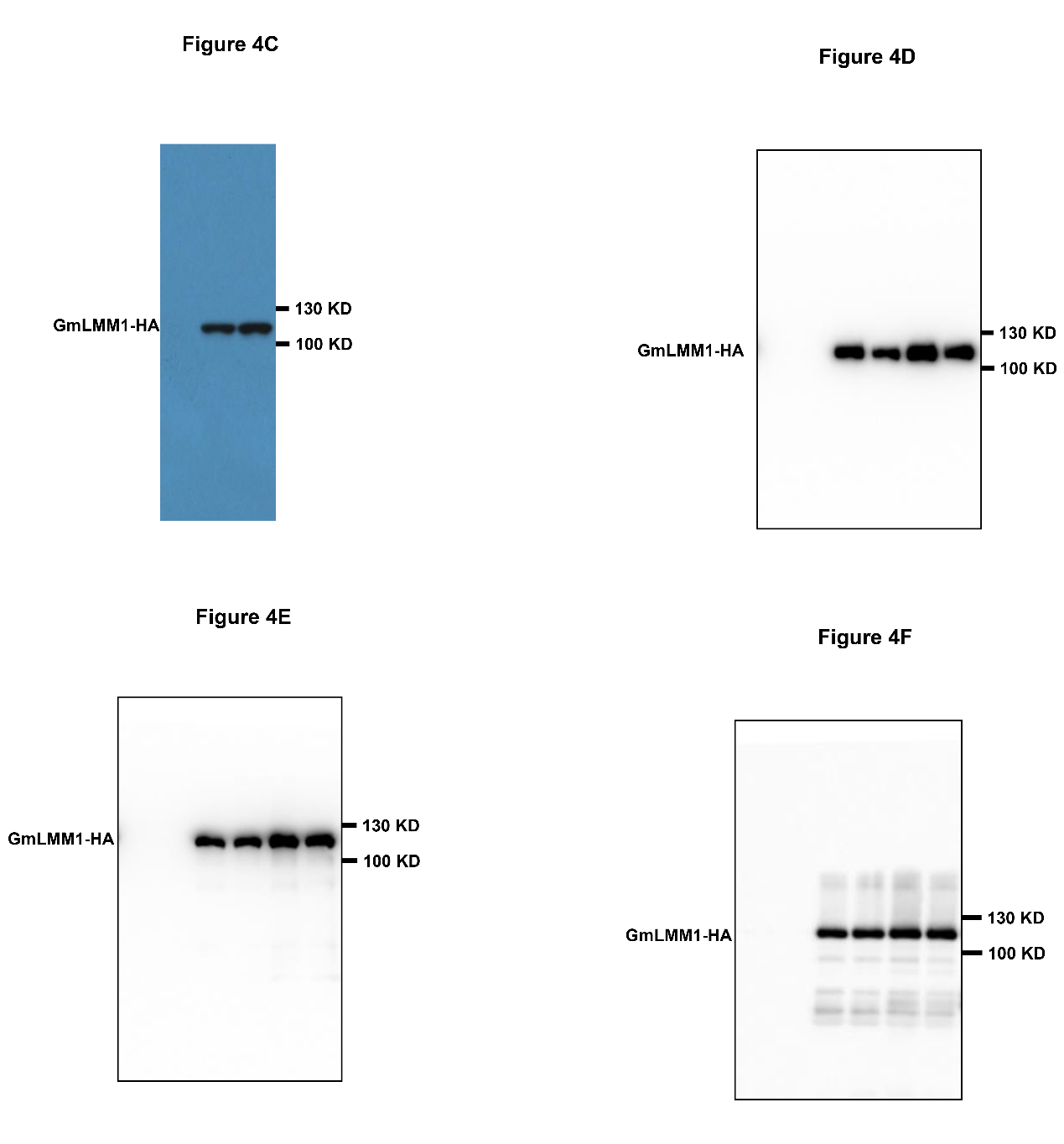

Supplement: Supplementary file 9 — Source Data for Figure 4 [file EMBR-21-e50442-s007.zip › Source Data for Figure 4/Source Data for Figure 4C-F.docx]

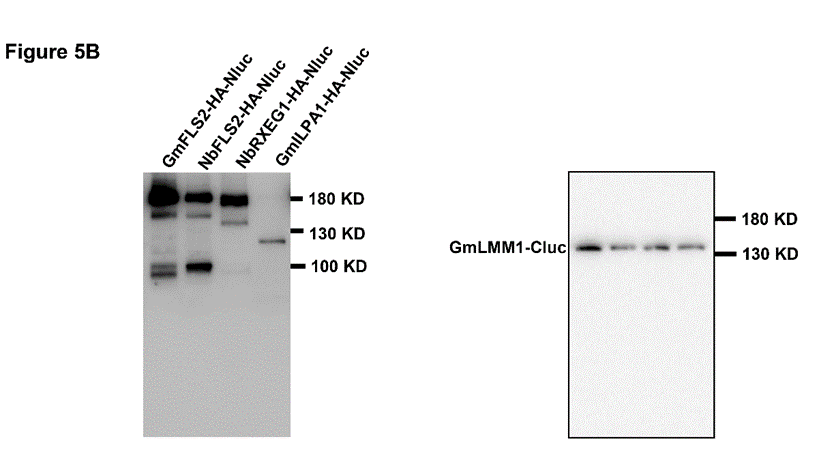


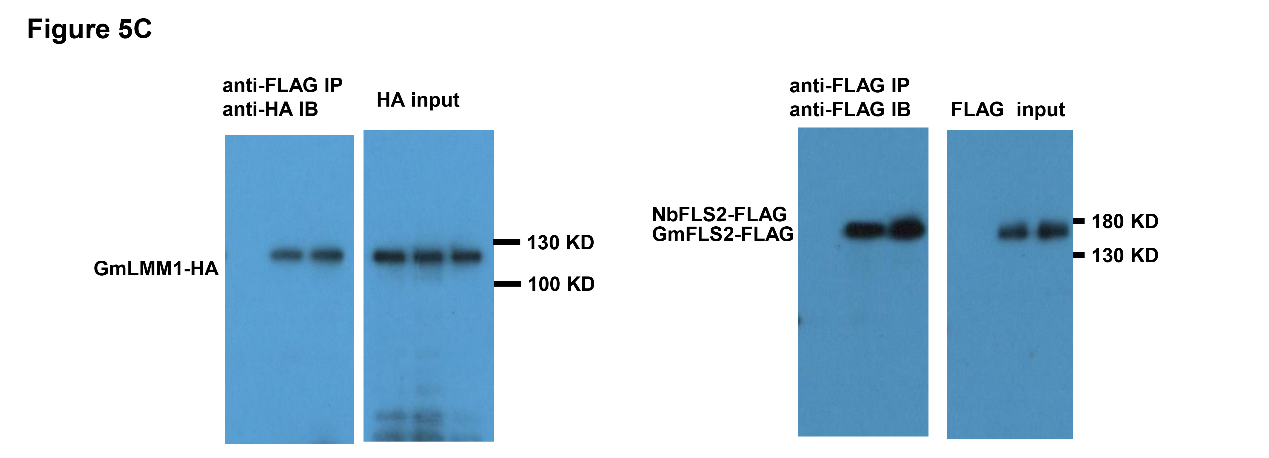

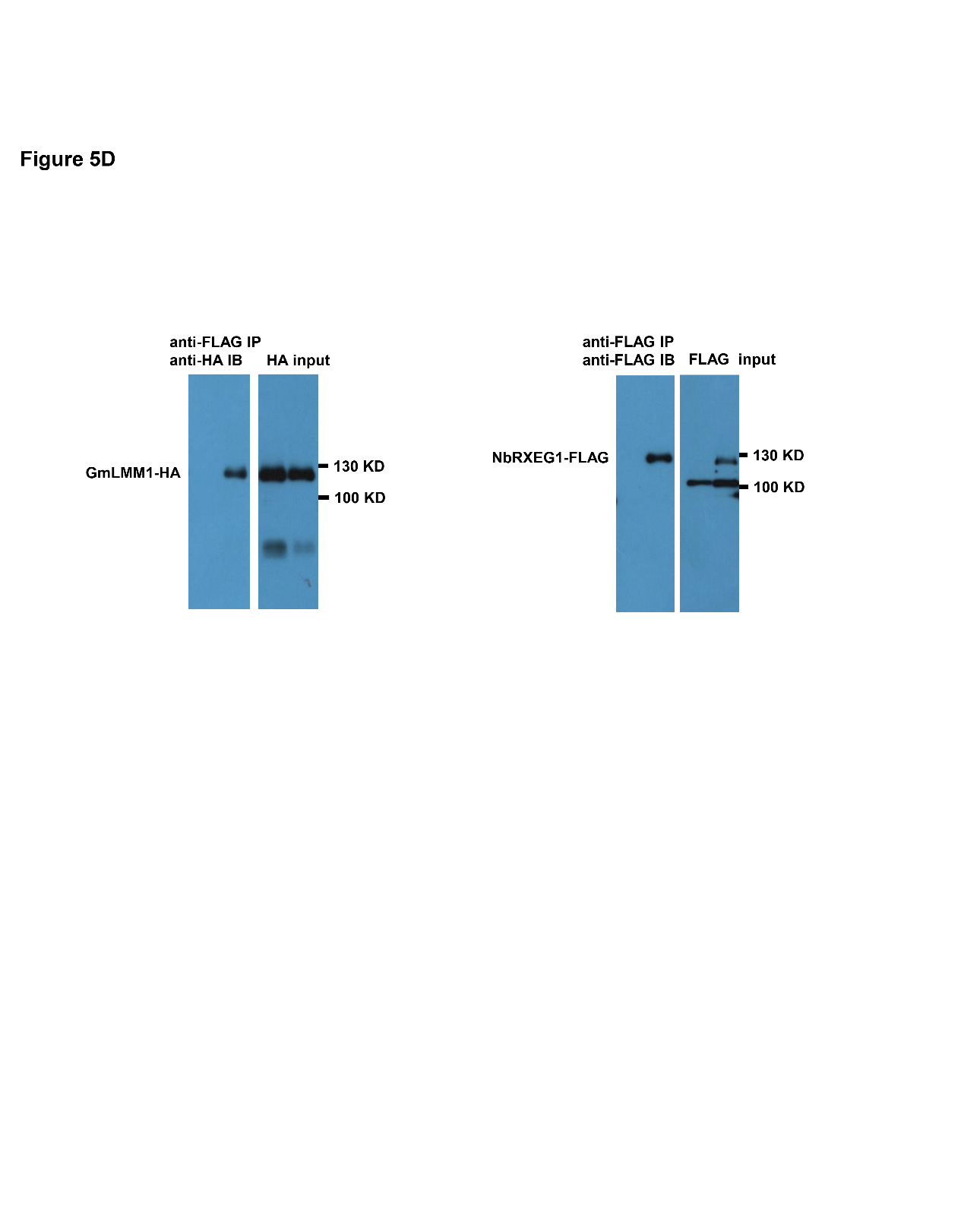


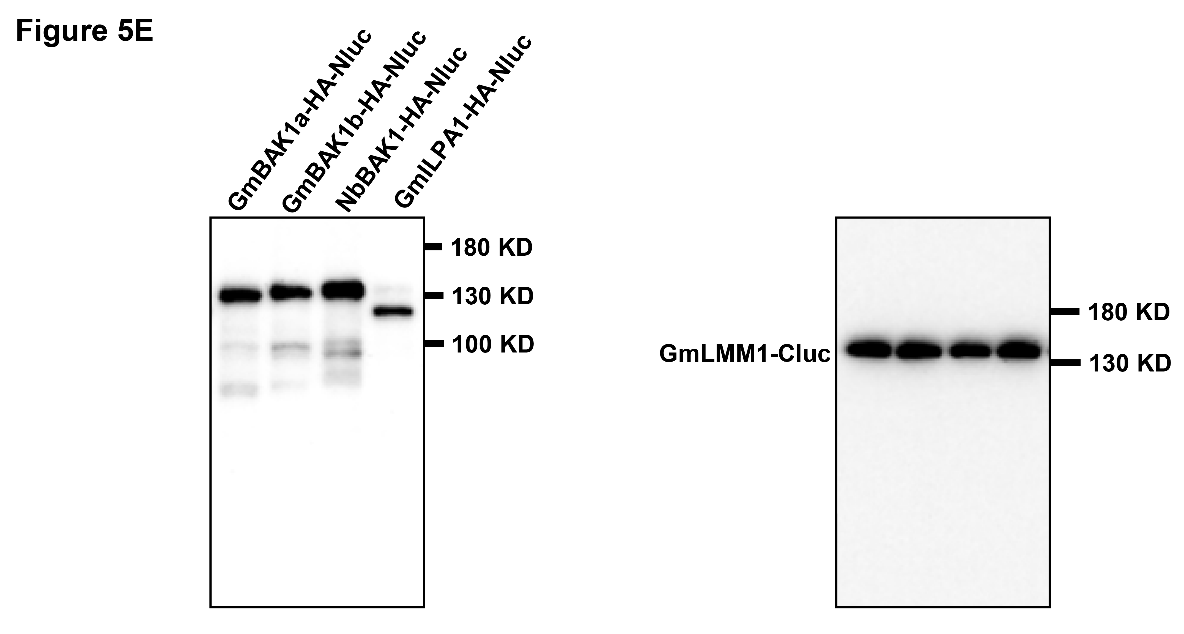


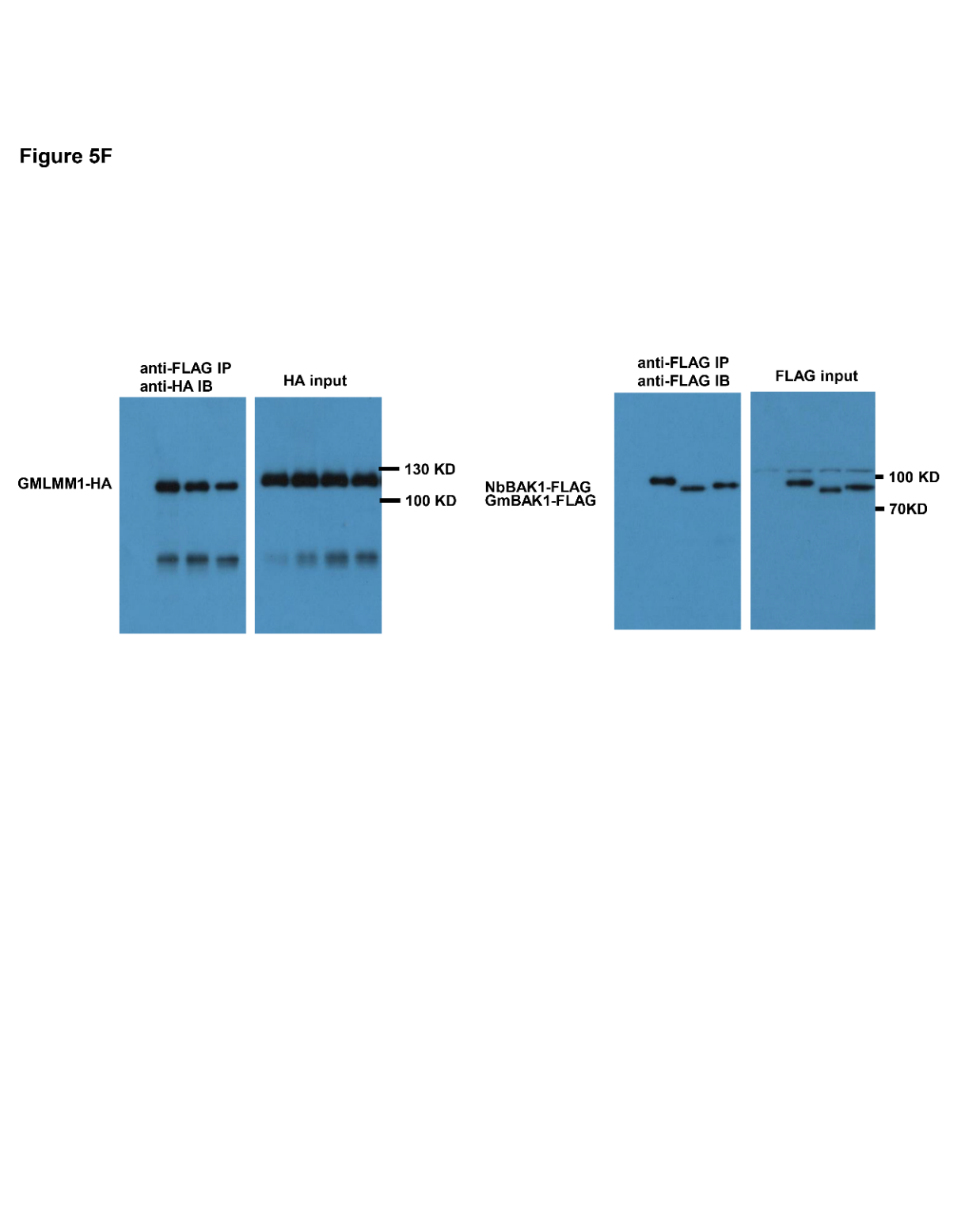

Supplement: Supplementary file 10 — Source Data for Figure 5 [file EMBR-21-e50442-s008.zip › Source Data for Figure 5/Source Data for Figure 5B-F.docx]

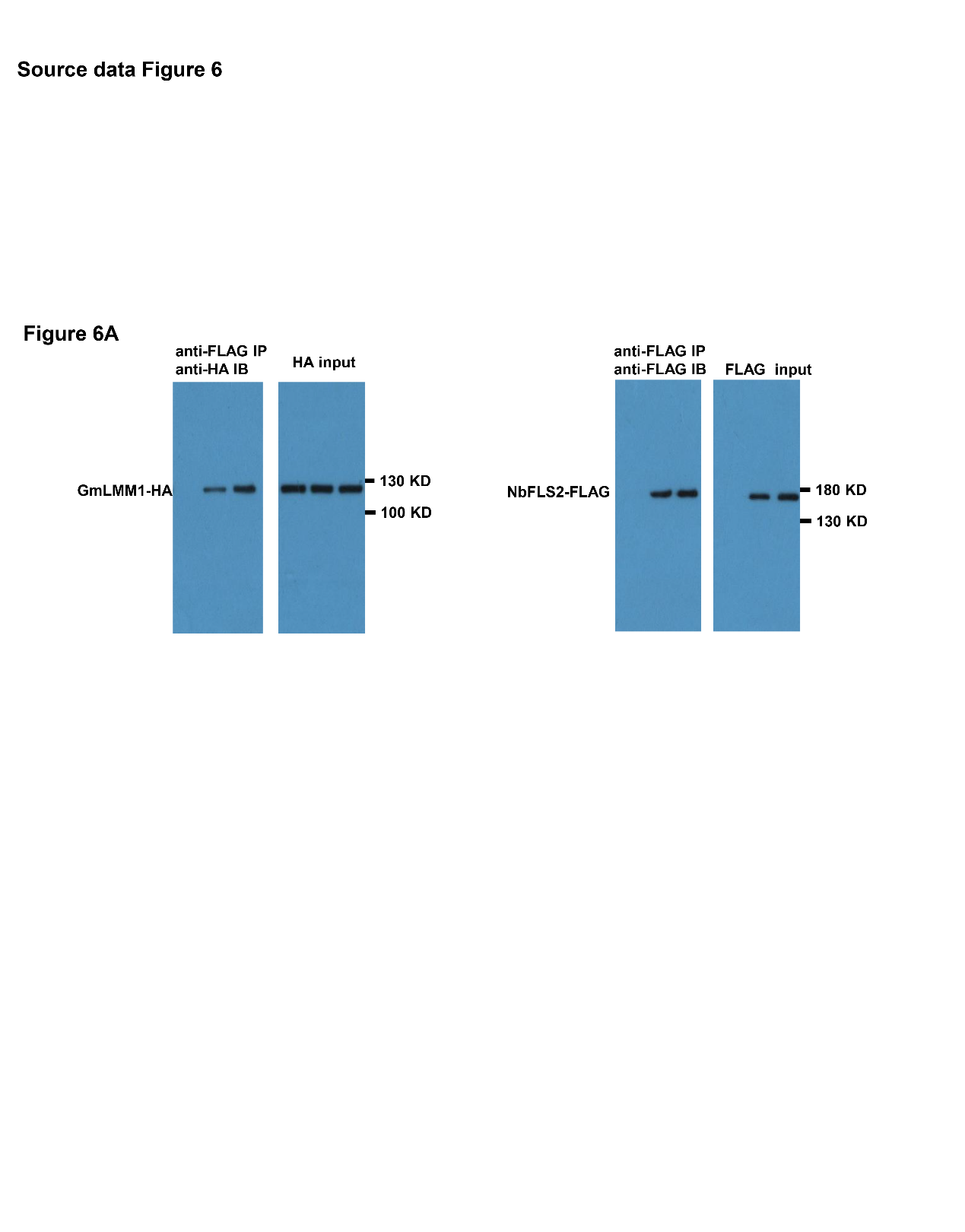

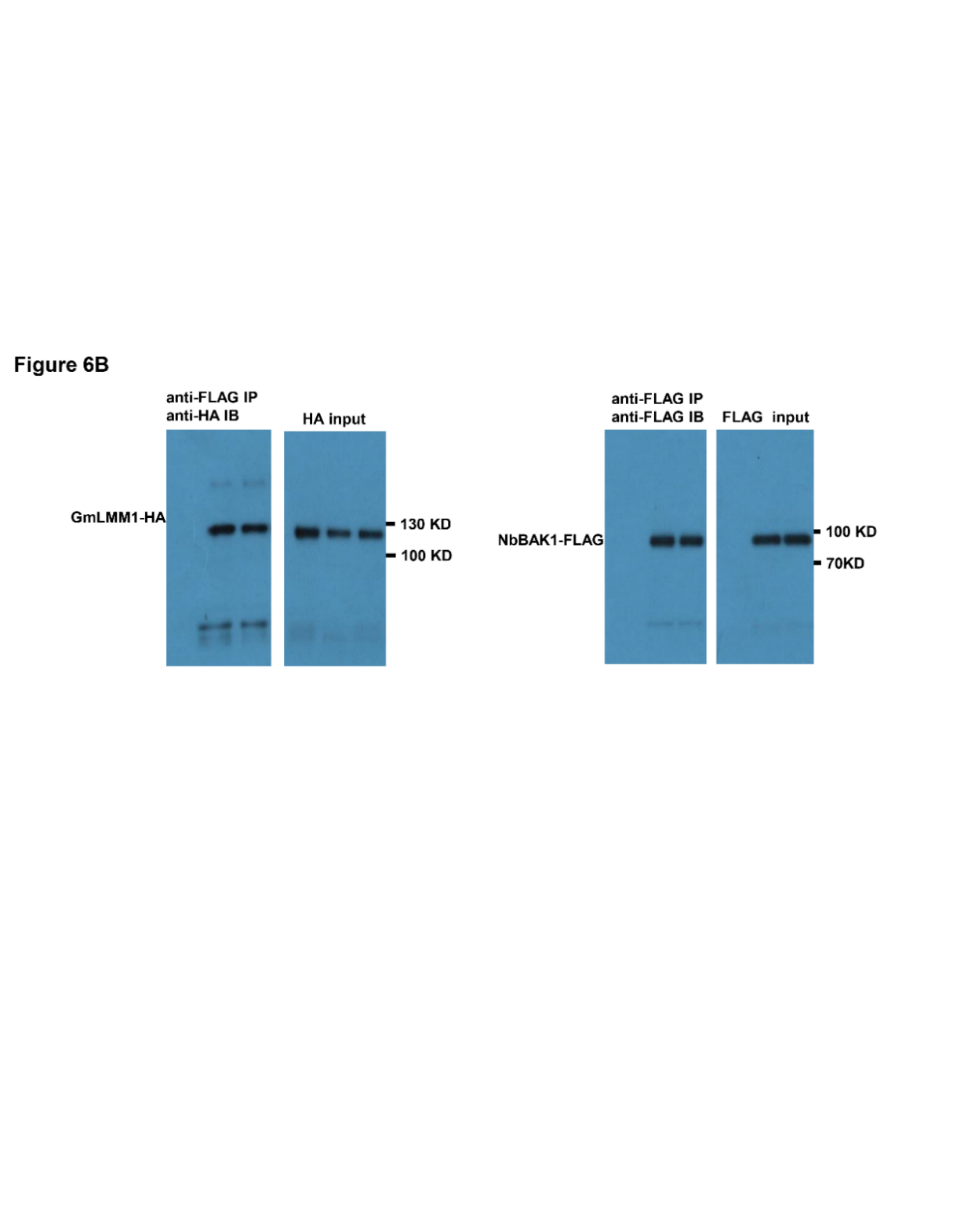

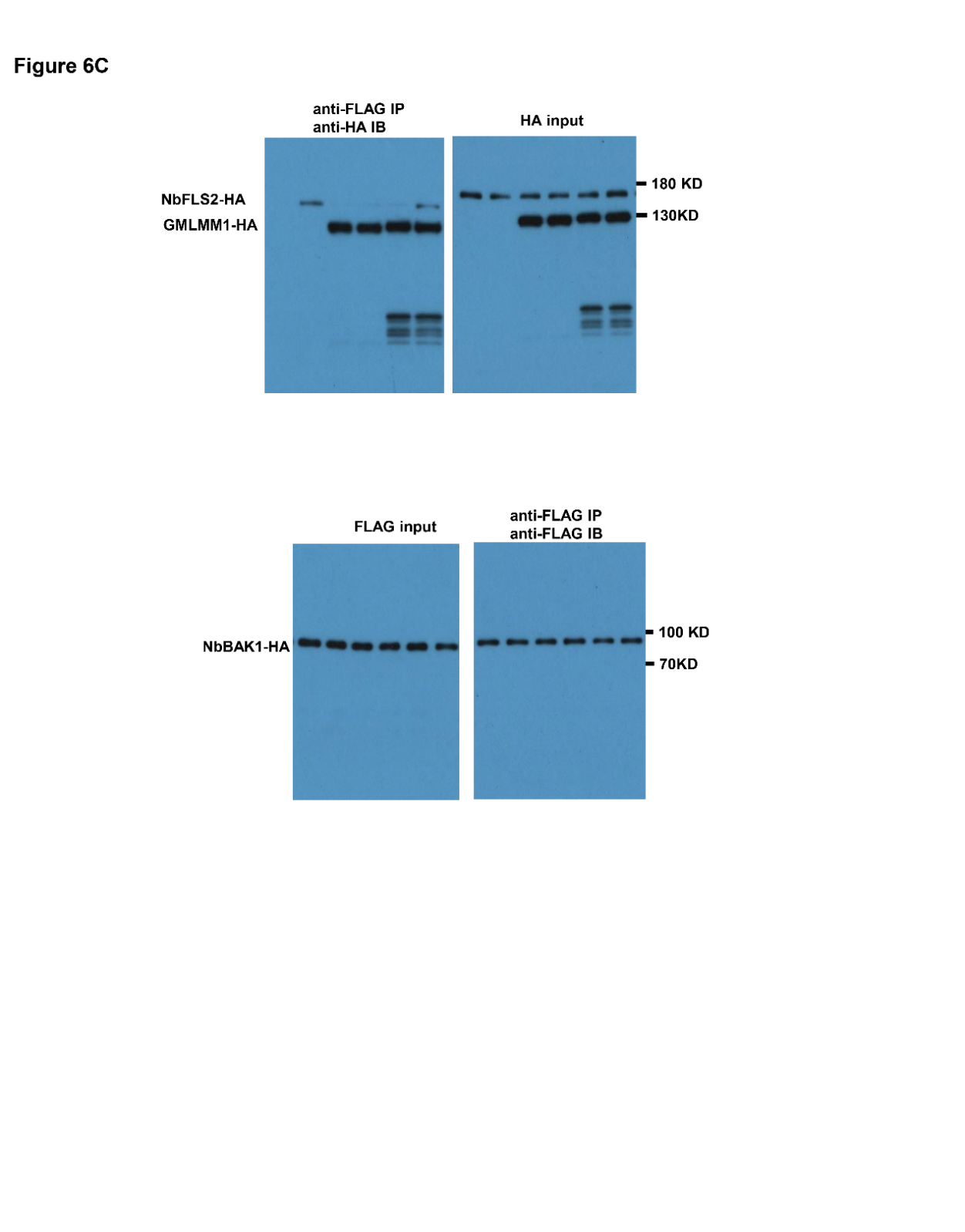

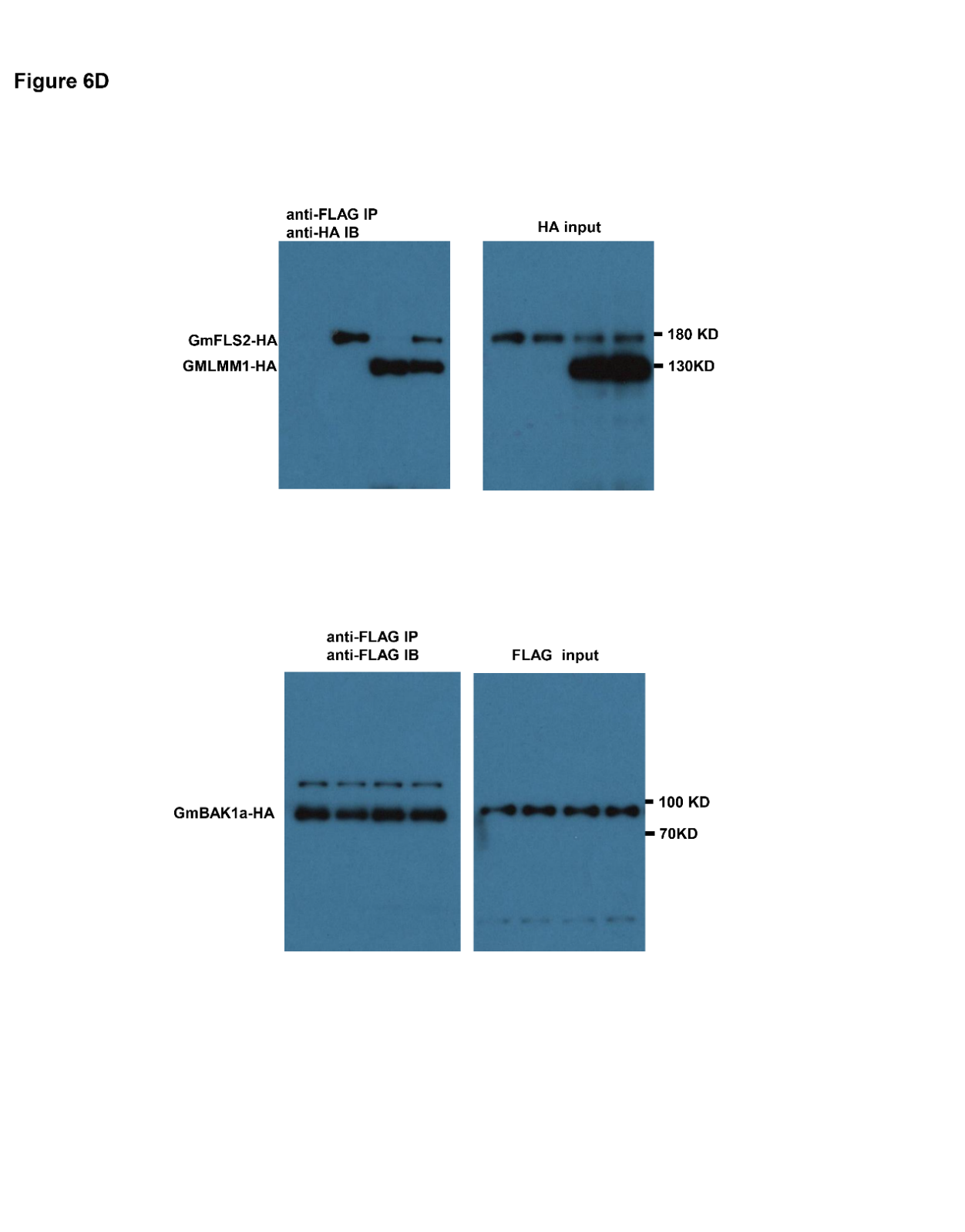

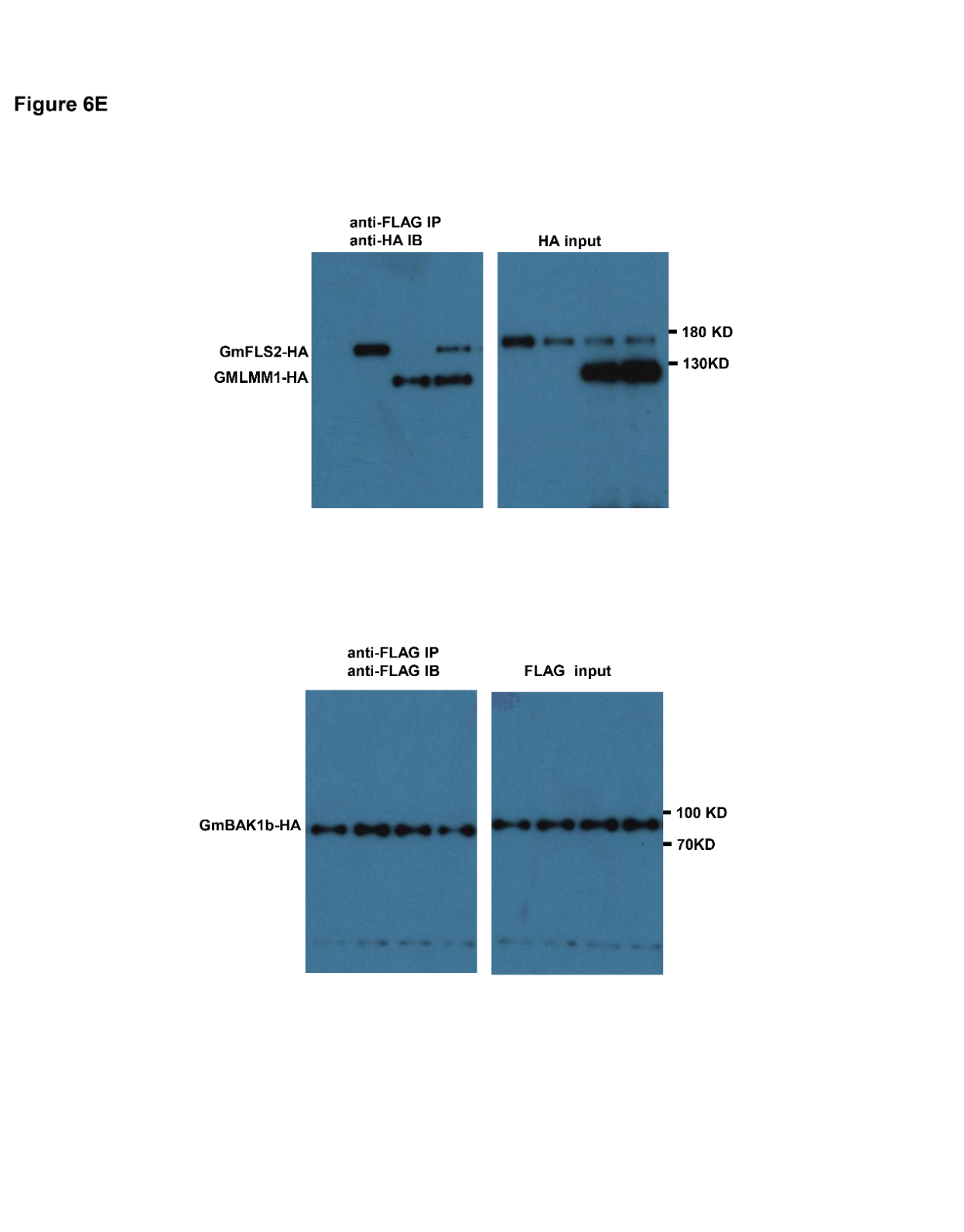

Supplement: Supplementary file 11 — Source Data for Figure 6 [file EMBR-21-e50442-s009.docx]
